# 非小细胞肺癌免疫治疗生物标志物的研究进展

**DOI:** 10.3779/j.issn.1009-3419.2021.102.40

**Published:** 2021-11-20

**Authors:** 川 黄, 雪 杨

**Affiliations:** 1 100730 北京，北京医院胸外科，国家老年医学中心，中国医学科学院老年医学研究院 Department of Thoracic Surgery, Beijing Hospital, National Center of Gerontology, Institute of Geriatric Medicine, Chinese Academy of Medical Sciences, Beijing 100730, China; 2 100142 北京，胸部肿瘤内一科，北京大学肿瘤医院暨北京市肿瘤防治研究所，恶性肿瘤发病机制及转化研究教育部重点实验室 Key Laboratory of Carcinogenesis and Translational Research (Ministry of Education/Beijing), Department I of Thoracic Oncology, Peking University Cancer Hospital & Institute, Beijing 100142, China

**Keywords:** 肺肿瘤, 免疫治疗, 生物标志物, Lung neoplasms, Immunotherapy, Biomarker

## Abstract

肺癌是全球死亡率最高的恶性肿瘤，非小细胞肺癌（non-small cell lung cancer, NSCLC）是其最常见的病理类型。近年来，免疫治疗给NSCLC的治疗带来划时代的变革，尤其是针对程序性死亡受体1（programmed cell death 1, PD-1）/程序性死亡配体1（programmed cell death ligand 1, PD-L1）的免疫检查点抑制剂（immune checkpoint inhibitors, ICIs），目前已被批准用于转移性NSCLC或部分局部晚期NSCLC患者的一线治疗和二线治疗。然而，仅15%-30%晚期NSCLC患者可以从免疫治疗中获得持续缓解和长期生存，如何探寻良好的生物标志物来有效地预测免疫治疗的疗效是当前面临的巨大挑战之一。本文对NSCLC免疫治疗疗效预测生物标志物的研究进展进行综述。

## Abstract (en)

Lung cancer is a malignant tumor with the highest mortality in the world. The most common pathological type of lung cancer is non-small cell lung cancer (NSCLC). In recent years, immunotherapy has brought about epoch-making changes in the treatment of NSCLC. In particular, immune checkpoint inhibitors (ICIs) targeting programmed cell death 1 (PD-1)/programmed cell death ligand 1 (PD-L1) have been approved for first-line and second-line treatment in patients with metastatic NSCLC. However, only 15% to 30% of patients with advanced NSCLC can achieve sustained remission and long-term survival from immunotherapy. Therefore, biomarker for predicting the efficacy of immunotherapy is particularly important. This article reviews the relevant literatures on predictive biomarkers for immunotherapy of NSCLC and provides direction for future research.

肺癌是全球死亡率最高的恶性肿瘤，国家癌症中心公布的2015年我国肺癌发病率达57.26/10万，死亡率达45.87/10万，位居所有恶性肿瘤之首^[1]^，其中非小细胞肺癌（non-small cell lung cancer, NSCLC）是最常见的病理类型。免疫治疗给NSCLC的治疗带来划时代的变革，针对程序性死亡受体1（programmed cell death 1, PD-1）/程序性死亡配体1（programmed cell death ligand 1, PD-L1）的免疫检查点抑制剂（immune checkpoint inhibitors, ICIs）已被批准用于转移性NSCLC或部分局部晚期NSCLC患者的一线和二线治疗。

然而，仅15%-30%的晚期NSCLC患者可以从免疫治疗中获得持续缓解和长期生存^[2]^，免疫治疗疗效预测指标仍在探索当中。2020年《*Immunity*》杂志提出的免疫治疗十大挑战就包括通过综合生物标志物来实现最大化的个体治疗突破^[3]^，而2021年美国临床肿瘤学会（American Society of Clinical Oncology, ASCO）发布的肿瘤进展年度报告更是确定策略，要更好地预测患者对免疫疗法的反应和耐药、识别与免疫治疗相关的血液和组织生物标志物^[4]^。因此深入了解肿瘤免疫全景，综合多种手段探寻预测性生物标志物是抗肿瘤免疫治疗面临的巨大挑战之一。NSCLC免疫治疗生物标志物包括免疫治疗疗效预测相关生物标志物、免疫治疗耐药/疾病进展/复发模式预测相关生物标志物以及免疫相关不良反应预测相关生物标志物。因篇幅所限，本文将重点探讨免疫治疗疗效预测相关生物标志物。

## 1 PD-L1

PD-L1是表达在肿瘤细胞及抗原呈递细胞上的蛋白，可以对适应性肿瘤免疫进行负向调节，使肿瘤细胞逃避免疫监视，是目前应用最广、证据最多的免疫治疗正性疗效预测生物标志物。美国国家综合癌症网络（National Comprehensive Cancer Network, NCCN）NSCLC指南自2019年起，PD-L1检测就由2A类上升至1类推荐，与表皮生长因子受体（epidermal growth factor receptor, EGFR）、间变性淋巴瘤激酶（anaplastic lymphoma kinase, ALK）、*c*-*ros*原癌基因1酪氨酸激酶（*c*-*ros* oncogene 1 receptor tyrosine kinase, ROS1）等生物标志物检测具有同等地位。应用免疫组织化学方法，PD-L1在60%的晚期NSCLC中有≥1%的肿瘤细胞表达，在25%-30%的患者中有高水平的表达（≥50%的肿瘤细胞）^[5-7]^，然而由于检测抗体和分析平台的差异，导致不同的研究之间存在显著差异。

## 2 PD-L1作为晚期NSCLC一线前瞻性分子标志物

### 2.1 PD-L1在晚期NSCLC免疫单药的疗效预测作用

KEYNOTE-024研究^[8]^吹响了免疫治疗进军NSCLC一线治疗的号角，开创了NSCLC免疫治疗新格局。这是一项国际化、随机、III期临床研究，评估了帕博利珠单抗对比铂类为基础的化疗用于未经治疗PD-L1肿瘤比例评分（tumor proportional score, TPS）≥50%且无*EGFR*突变或*ALK*易位的晚期NSCLC患者。截至2020年6月1日，经过5年长期随访，帕博利珠单抗组较化疗组中位无进展生存期（progression-free survival, PFS）延长（7.7个月*vs* 5.5个月，HR=0.50，95%CI：0.39-0.65），帕博利珠单抗组5年总生存（overall survival, OS）率较化疗组提升近1倍（31.9% *vs* 16.3%），中位OS明显延长（26.3个月*vs* 13.4个月，HR=0.62，95%CI：0.48-0.81）^[9]^。而KEYNOTE-042研究^[10]^更是把目标人群扩展到PD-L1表达≥1%的NSCLC人群，但在此研究中47%患者PD-L1 TPS为50%或更高，而这部分PD-L1高表达的人群是免疫治疗生存获益主要人群。IMpower 110研究^[11]^同样提示阿替利珠单抗较化疗仅能改善PD-L1高表达患者的预后。此外，在一些回顾性研究及2021年ASCO均报道对于PD-L1表达≥50%的人群可进一步细化，在更高表达的人群当中疗效更优^[12]^。因此，对于PD-L1高表达人群进一步细化是未来研究的方向。

然而在其他PD-1/PD-L1单抗一线单药研究中，包括CheckMate-026^[13]^、MYSTIC研究^[14]^，并未发现在PD-L1阳性患者中应用免疫治疗较化疗有生存率提高。为何会出现此种差异？究其原因，包括研究入组标准不同、应用免疫治疗具体药物不同、PD-L1检测抗体及平台不同、更重要的是纳入人群PD-L1表达水平差异。在这些临床研究中，PD-L1表达水平按照为达到试验研究阳性统计学结果进行人为预设，而不是像NSCLC驱动基因靶向治疗（如*EGFR*突变、*ALK*易位）一样反映肿瘤独特的生物学特性，这可能是PD-L1作为生物标志物没有驱动基因阳性指导后续治疗效果明确原因之一。

### 2.2 PD-L1在晚期一线NSCLC免疫联合治疗的疗效预测作用

免疫联合治疗模式包括免疫联合化疗、双免疫治疗、免疫联合抗血管治疗以及双免疫联合化疗等策略。

#### 2.2.1 PD-L1对免疫联合化疗的疗效预测作用

PD-L1表达并不能预测免疫联合化疗的疗效。KEYNOTE-189研究^[15]^是帕博利珠单抗联合化疗用于晚期非鳞NSCLC一线治疗的首个III期研究，该研究提示培美曲塞-铂类一线治疗转移性非鳞状NSCLC患者时，与联合安慰剂相比，联合帕博利珠单抗可显著改善OS和PFS，且不受肿瘤PD-L1表达情况的影响。在亚组分析中，PD-L1 TPS≥50%、TPS 1%-49%以及TPS < 1%这三组中，均观察到相较于单纯化疗帕博利珠单抗联合化疗对OS的一致获益（HR=0.59, 95%CI: 0.40-0.86; HR=0.66, 95%CI: 0.46-0.96; HR=0.51, 95%CI: 0.36-0.71）。IMpower130^[16]^、KEYNOTE-407研究^[17]^也得出类似结论。

#### 2.2.2 PD-L1对多个免疫药物联合治疗的疗效预测作用

在双免疫治疗方面，CheckMate-227研究提示无论在PD-L1≥1%人群还是PD-L1 < 1%的人群，纳武利尤单抗联合低剂量伊匹木单抗组的2年OS率均为40%，提示无论PD-L1表达与否，均能从双免疫治疗中获益^[18]^。

而PD-L1表达在一些新型免疫靶点药物，如T细胞免疫球蛋白和ITIM结构域蛋白（T cell immunoreceptor with Ig and ITIM domains, TIGIT）中的预测作用值得期待。TIGIT是脊髓灰质炎病毒受体（poliovirus receptor, PVR）家族成员，主要在活化T细胞和自然杀伤（natural killer, NK）细胞表面表达。TIGIT通过与肿瘤细胞上表达的PVR结合，抑制细胞毒性T细胞和NK细胞介导的肿瘤杀伤作用。因此，同时抑制TIGIT及PD-1能够协同免疫细胞杀伤肿瘤，增强抗肿瘤免疫应答。2020年ASCO年会报道的CITYSCAPE研究，评估TIGIT抗体Tiragolumab联合阿替利珠单抗对比单药阿替利珠单抗一线治疗NSCLC的疗效，结果发现在PD-L1 TPS≥50%的患者中，联合治疗组与单药阿替利珠单抗的客观缓解率（objective response rate, ORR）分别为66%和24%。而在PD-L1 TPS 1%-49%的人群中两组ORR无差异（分别为16%和18%）。PFS方面同样发现在PD-L1 TPS≥50%的患者中，联合治疗组更能获益（HR=0.30, 95%CI: 0.15-0.61）。而在PD-L1 TPS 1%-49%的人群中并未发现联合治疗组与单药组的PFS差异^[19]^。

其他新型的靶点药物包括Eftilagimod alpha（可溶性LAG-3蛋白）^[20]^与PD-1单抗联合、抗TIM-3抗体LY3321367^[21]^与PD-L1单抗联合以及M7824靶向PD-L1通路和TGF-β通路的双功能抗体^[22]^在NSCLC的研究也在如火如荼地进行，目前暂无明确的生物标志物。

#### 2.2.3 PD-L1对免疫联合抗血管治疗的疗效预测作用

IMpower150研究^[23]^是一项非鳞NSCLC患者一线治疗的随机对照III期临床试验，旨在评估阿替利珠单抗+卡铂+紫杉醇联合或不联合贝伐珠单抗的疗效及安全性。该研究评估阿替利珠单抗+贝伐珠单抗+卡铂+紫杉醇（ABCP组）和阿替利珠单抗+卡铂+紫杉醇（ACP组）分别对贝伐珠单抗+卡铂+紫杉醇（BCP组），一线治疗晚期非鳞NSCLC的OS结果，并对PD-L1表达状态进行了亚组分析。研究发现无论患者PD-L1的表达状态如何，ABCP组较BCP组的PFS（8.3个月*vs* 6.8个月，HR=0.62，95%CI：0.52-0.74，*P* < 0.001）和OS（19.2个月*vs* 14.7个月，HR=0.78，95%CI：0.64-0.96，*P*=0.02）均有不同程度的改善，亚组分析显示，PD-L1高表达、PD-L1低表达（< 50%肿瘤细胞表达PD-L1）、PD-L1阴性（< 10%肿瘤浸润免疫细胞表达PD-L1）三组患者获益的HR值依次升高（HR=0.39, 95%CI: 0.25-0.60; HR=0.56, 95%CI: 0.41-0.77; HR=0.77, 95%CI: 0.61-0.99），提示PD-L1高表达患者接受免疫联合抗血管治疗及化疗的疗效更佳。免疫联合抗血管在NSCLC一线治疗中除了IMpower150外仅有一些小样本研究，如一线晚期NSCLC信迪利单抗联合安罗替尼的研究^[24]^，PD-L1表达与免疫联合抗血管治疗疗效预测的关系尚不明确。

#### 2.2.4 PD-L1对双免疫联合化疗的疗效预测作用

CheckMate-9LA研究是目前唯一报道研究结果的双免疫联合化疗2个周期后随后应用双免疫治疗的III期研究，结果提示纳武单抗联合伊匹木单抗及2个周期的同步化疗可以延长患者的OS，且无论患者PD-L1表达水平和肿瘤组织学类型如何，所有疗效评估均显示出双免疫联合化疗的一致临床获益^[25]^。

#### 2.2.5 PD-L1对免疫治疗联合放化疗的预测作用

PACIFIC研究奠定了度伐利尤单抗作为III期不可手术切除、接受初始放化疗的治疗后无进展的局部NSCLC患者维持治疗的基础。与安慰剂组相比，度伐利尤单抗显著延长PFS（16.8个月*vs* 5.6个月，HR=0.52，95%CI：0.42-0.65）。而PD-L1表达作为提前预设的分层因素，据此进行的PFS、OS亚组分析发现PD-L1低表达人群免疫巩固治疗后并无PFS及OS获益^[26]^，提示同步放化疗后的免疫巩固治疗仍需更“精准”，而PD-L1表达可较好地对获益人群进行筛选。

### 2.3 PD-L1作为晚期NSCLC后线生物标志物

KEYNOTE-010研究探索了帕博利珠单抗后线治疗PD-L1≥1%的晚期NSCLC患者的疗效及安全性，结果显示PD-L1≥50%组和PD-L1≥1%组PFS疾病进展风险分别降低43%和27%，3年PFS率分别为21.9%和12.7%，3年OS率分别为34.5%和22.9%，提示PD-L1能够较好的预测帕博利珠单抗后线治疗的疗效^[27]^。

值得注意的是，KEYNOTE-010研究最初对于PD-L1的检测是用初次病理组织检测还是复发/转移后新取得病理组织进行检测并未做要求，后续方案修改后要求应用帕博利珠单抗治疗前重新进行活检的标本行PD-L1的检测。后续方案修改的原因可能与化疗会影响PD-L1的表达有关，而KEYNOTE-010研究结果^[28]^提示治疗后再次活检组织的PD-L1表达可预测帕博利珠单抗后线单药治疗的疗效。然而，CheckMate-017、CheckMate-057^[29]^及OAK研究^[30]^中并未对检测PD-L1标本取得时机做明确要求，这两项研究并未发现PD-L1表达与预后的相关性，提示PD-L1表达对于免疫后线治疗的预测作用有限。

### 2.4 PD-L1作为晚期NSCLC生物标志物的局限性

PD-L1可以预测一线免疫治疗单药的疗效，但在免疫联合治疗及后线单药治疗中预测作用有限。究其原因，可能与化疗可上调肿瘤免疫原性相关，而抗血管治疗与免疫治疗联合可能通过正常化肿瘤血管结构促进T细胞浸润、降低了髓源性抑制细胞和调节性T细胞的活性，重塑肿瘤微环境等机理增加免疫治疗疗效。在联合治疗的模式下，肿瘤微环境的变化更为复杂，单一靠PD-L1不太可能完美预测联合治疗的疗效。

PD-L1作为免疫治疗疗效预测生物标志物并不完美，主要表现在以下几个方面：PD-L1在组织内表达具有肿瘤内异质性、抗肿瘤治疗可能会改变肿瘤组织内PD-L1表达、部分患者无法获取组织标本、检测受到组织标本的固定及保存方法等多种因素影响、不同PD-L1检测抗体和平台、对PD-L1表达判读标准的不一致。因此进一步标化、简化PD-L1检测流程以有效指导患者治疗迫在眉睫。而检测可溶性PD-L1、循环肿瘤细胞上PD-L1表达^[31]^可能成为一种无创、安全、准确且能预测疗效和动态监测的技术，也是未来值得研究的方向。

## 3 肿瘤突变负荷（tumor mutational burden, TMB）

TMB是指特定基因组区域内体细胞非同义突变的个数，通常用每兆碱基的突变数量（mut/Mb）表示，是一种定量的生物标志物。TMB可能间接反映肿瘤新抗原水平，而新抗原被认为可以协助免疫系统识别肿瘤并刺激T细胞的增生和抗肿瘤反应。2014年TMB与免疫治疗的初次邂逅缘起于黑色素瘤^[32]^。而根据KEYNOTE-158结果^[33]^，2020年TMB经FDA批准成为泛实体瘤免疫治疗生物标志物。大量研究以及*meta*分析^[34-36]^表明，TMB与PD-1/PD-L1抑制剂在多个瘤种中的疗效相关。其中一项来自中国学者的镜像荟萃分析^[37]^提示在接受免疫治疗的NSCLC患者中，高TMB亚组的PFS显著优于低TMB亚组（HR=0.56, 95%CI: 0.47-0.67），并且在不同治疗路线、方案模式、TMB检测方法和PD-L1表达的亚组分析中一致。

### 3.1 TMB对免疫单药的疗效预测作用

TMB与免疫单药疗效相关。在KEYNOTE-042和KEYNOTE-010中研究中较高的组织TMB（tissue TMB, tTMB）与帕博利珠单抗单独用药组的疗效改善相关，但与化疗无关。帕博利珠单抗对比化疗的OS中位数如下所示：KEYNOTE-042中tTMB≥175 mut/外显子组亚组：21.9个月和11.6个月（HR=0.62, 95%CI: 0.48-0.80），而tTMB < 175 mut/外显子组亚组：12.0个月和12.3个月（HR=1.09, 95%CI: 0.88-1.36）；KEYNOTE-010中tTMB≥175 mut/外显子组亚组：14.1个月和7.6个月（HR=0.56, 95%CI: 0.38-0.83），而TMB < 175 mut/外显子组亚组：9.3个月和7.2个月（HR=0.85, 95%CI: 56-1.30）^[38]^。

### 3.2 TMB对双免疫治疗的疗效预测作用

CheckMate-227研究^[39]^提示TMB≥10 mut/Mb的晚期NSCLC一线纳武单抗联合伊匹木单抗治疗，与化疗相比，PFS得到显著改善，且无需考虑PD-L1表达状态，但在TMB≥10 mut/Mb组和TMB < 10 mut/Mb组之间并未发现OS差异（HR=0.77, 95%CI: 0.56-1.06; HR=0.78, 95%CI: 0.61-1.00）。与CheckMate-227研究结果类似，CheckMate-9LA同样发现tTMB和血液TMB（blood TMB, bTMB）数值更高的患者，ORR和PFS获益更多，但未发现OS差异^[40]^。而在CheckMate-227研究中OS阴性原因可能与化疗组进展后二线交叉到免疫治疗组有关。值得注意的是，在CheckMate-227研究中，有42%标本不符合TMB检测要求，所以对该研究的结果解读需慎重。上述结果提示对于双免疫治疗来说，TMB可能是一个预测因素，而不是预后因素。

### 3.3 TMB对免疫联合化疗的疗效预测作用

KEYNOTE-021、KEYNOTE-189和KEYNOTE-407研究均未发现TMB与免疫联合化疗疗效的相关性^[41]^。

### 3.4 TMB作为晚期NSCLC生物标志物的局限性

TMB是很有潜力的生物标志物，目前存在的问题在于对不同的瘤种和不同PD-1/PD-L1单抗TMB预测值差异较大，不同检测平台和检测基因数将会影响TMB结果。TMB与PD-L1无明显相关性^[42]^。因此TMB在免疫单药和双免疫治疗中预测作用值得肯定，在免疫联合化疗预测作用有限，其在NSCLC免疫治疗中的预测价值值得进一步探讨和优化。

## 4 其他新兴生物标志物

### 4.1 微卫星不稳定性

微卫星不稳定性（microsatellite instability, MSI）已经广泛应用于结直肠癌患者的临床治疗，在肺癌中的应用较少，预测价值尚不明确，未来需要更多研究探索。

### 4.2 循环肿瘤DNA

循环肿瘤DNA（circulating tumor DNA, ctDNA）在肺癌早期诊断、术后肿瘤残留判断、肿瘤复发监测、驱动基因检测及靶向耐药机制探索方面都进行过较为深入的研究和探讨。近年来多项研究提示ctDNA水平的变化可预测免疫治疗的获益情况。

2020年《*Nature Cancer*》杂志上发表的研究提示基线ctDNA水平与免疫治疗OS、PFS和客观反应率（objective response rate, ORR）密切相关，基线ctDNA水平低者对免疫治疗呈现出更好的治疗效果；ctDNA的清除要较传统影像学变化更敏感，可作为独立于PD-L1、TMB的免疫治疗标记物^[43]^。但较为遗憾的是，此研究中并未包括NSCLC患者。而另外一项有关晚期NSCLC的研究^[44]^提示ctDNA及血浆蛋白组学的动态改变可能预测接受免疫单药或免疫联合化疗的患者预后。患者接受免疫单药/免疫联合化疗期间的ctDNA分子应答能够预测患者生存，并建立了对分子应答、复发及评估患者获益的整合预测模型。

### 4.3 评分预测模型

2021欧洲肺癌大会报道一项最新研究，通过构建两个评分系统（EPSILoN评分和NLCIPS评分）来预测NSCLC患者接受免疫治疗的疗效^[45]^。EPSILoN评分包括5个临床/血液参数，分别是美国东部肿瘤协作组体力状况（Eastern Cooperative Oncology Group performance status, ECOG PS）评分、吸烟、肝转移、乳酸脱氢酶（lactate dehydrogenase, LDH）和中性粒细胞淋巴细胞比值（neutrophil to lymphocyte ratio, NLR），根据患者对免疫治疗的反应将其分为良好、中等、较差三个组。另一项NLCIPS评分则是基于吸烟指数和NLR的疗效预测模型，根据1年无进展生存期把患者分为良好、中等、差、非常差四组。该研究结果显示EPSILoN和NLCIPS评分与免疫治疗疗效有显著相关性，有助于筛选能从免疫治疗中获益的潜在人群。

### 4.4 T细胞炎症基因表达谱（T-cell-inflamed gene expression profile, GEP）

Cristescu等^[46]^回顾来自4项KEYNOTE临床试验的22种肿瘤类型的300例晚期实体瘤和黑色素瘤患者样本，旨在评估TMB和T细胞炎症GEP单用或联合预测帕博利珠单抗单药疗效的作用。该研究根据预先设定的TMB和GEP界值，将患者分为四组：低GEP/低TMB、低GEP/高TMB、高GEP/低TMB和高GEP/高TMB。研究发现无论癌种，帕博利珠单抗单药用于高GEP/高TMB组患者的ORR及PFS更高，提示在多癌种患者队列中TMB及T细胞炎症GEP高表达与帕博利珠单抗单药治疗应答及预后更优相关。针对不同GEP/TMB表达晚期NSCLC一线行帕博利珠单抗+LAG-3单抗/仑伐替尼/CTLA-4抑制剂的II期研究KEYNOTE-495目前也在进行当中。

### 4.5 肿瘤浸润淋巴细胞（tumor-infiltrating lymphocytes, TILs）

近期发表的一项荟萃分析^[47]^提示肿瘤内部和间质的CD8^+^ T细胞高水平可预测多个瘤种中免疫治疗的疗效，包括免疫单药和免疫联合治疗。根据PD-L1表达和TILs将肿瘤微环境分为四种类型，分别是PD-L1（-）/TILs（-）肿瘤（I型）：免疫忽视型，免疫细胞不在肿瘤部位蓄积；PD-L1（+）/TILs（+）肿瘤（II型）：抗PD-L1疗法治疗更有效；PD-L1（-）/TILs（+）肿瘤（III型）：免疫耐受型，存在TILs，但无法诱导TME中的PD-L1表达；PD-L1（+）/TILs（-）肿瘤（IV型）：通过致癌途径内源性诱导PD-L1表达。上述分型在肿瘤发生机制及治疗中具有重要意义，I型、III型和IV型可通过不同的策略转化为II型。TILs不仅可作为一项生物标志物进行探索，还可能成为一种潜在的肿瘤治疗方法。

### 4.6 其他指标

免疫抑制的生物标志物，包括淋巴细胞活化基因3（lymphocyte activation gene 3, LAG-3）、调节性T细胞（regulatory T cells, Treg）、骨髓衍生抑制细胞（myeloid-derived suppressor cells, MDSCs）等。肿瘤基因组特征，正向基因包括*K*-*ras*原癌基因、*p53*抑癌基因、*PBRM1*基因等，负向基因包括*EGFR*、*ALK*、*PTEN*、丝氨酸/苏氨酸激酶11（serine/threonine kinase 11, *STK11*）等。此外还有患者胚系遗传因素[如人类白细胞抗原（human leukocyte antigen, HLA）I类多样性等]、宿主肠道微生物菌群多样性等生物标志物与NSCLC免疫治疗疗效的相关性也正在探索中。

## 5 结论

如何探寻良好的疗效预测生物标志物仍然是NSCLC免疫治疗面临的的重大挑战。PD-L1仍是目前免疫治疗的最重要生物标志物，PD-L1表达与免疫治疗疗效相关性在免疫单药方面表现更好，但在免疫联合治疗方面表现欠佳。而TMB作为预测性生物标志物作用仍面临挑战。新兴分子标志物发展如火如荼，前景值得期待。未来需要建设多维度生物标志物综合预测免疫治疗疗效的平台，积极探索更加精准、行之有效的生物标志物，用于指导临床治疗选择。
